# Adaptive Transcutaneous Power Transfer to Implantable Devices: A State of the Art Review

**DOI:** 10.3390/s16030393

**Published:** 2016-03-18

**Authors:** Kara N. Bocan, Ervin Sejdić

**Affiliations:** Department of Electrical and Computer Engineering, Swanson School of Engineering, University of Pittsburgh, Pittsburgh, PA 15213, USA; knb12@pitt.edu

**Keywords:** wireless power transfer, transcutaneous energy transfer, implantable medical devices, adaptive, tuning

## Abstract

Wireless energy transfer is a broad research area that has recently become applicable to implantable medical devices. Wireless powering of and communication with implanted devices is possible through wireless transcutaneous energy transfer. However, designing wireless transcutaneous systems is complicated due to the variability of the environment. The focus of this review is on strategies to sense and adapt to environmental variations in wireless transcutaneous systems. Adaptive systems provide the ability to maintain performance in the face of both unpredictability (variation from expected parameters) and variability (changes over time). Current strategies in adaptive (or tunable) systems include sensing relevant metrics to evaluate the function of the system in its environment and adjusting control parameters according to sensed values through the use of tunable components. Some challenges of applying adaptive designs to implantable devices are challenges common to all implantable devices, including size and power reduction on the implant, efficiency of power transfer and safety related to energy absorption in tissue. Challenges specifically associated with adaptation include choosing relevant and accessible parameters to sense and adjust, minimizing the tuning time and complexity of control, utilizing feedback from the implanted device and coordinating adaptation at the transmitter and receiver.

## 1. Introduction

Implantable medical devices have become a huge market, with over 20 million individuals estimated to have an implanted medical device and over $300 billion in associated costs in the U.S. in 2000. Over one million patients in the U.S. have cardiac pacemakers; 250,000 new pacemakers are implanted each year; 100,000 implantable cardioverter defibrillators (ICDs) are implanted each year; and 120,000 patients in the U.S. have cochlear implants [1].

The term “implantable medical device” has been used to encompass many devices, from pacemakers to orthopedic implants to heart valves. For the purposes of this work, “implantable medical device” will refer to any implanted device that requires electrical power: for example, to stimulate the atrial node in the case of a pacemaker or to power a small microprocessor in the case of an implanted sensing device.

Fully implantable medical devices have already effected vast improvements in patient monitoring and treatment by eliminating percutaneous cables that are prone to infection and limit patient mobility. These include continuous therapy devices, such as the implantable cardioverter/defibrillator (ICD), electronic pacemaker, implantable neurostimulators and fully-implantable drug delivery pumps [2,3,4,5,6].

The benefits of implantable devices also include prostheses, such as the cochlear implant and retinal implant [7,8,9]. Cortical implant research promises neural control of movement prosthetics and the valuable inclusion of sensory feedback [1]. Implantable electrodes have been developed for nerve and muscle stimulation [1].

Implantable sensing devices are particularly important in situations where the biological signals to be accessed are inside the body and cannot be reliably sensed non-invasively [10]. This includes wireless implantable biosensors, such as those used with insulin pumps, although commercial sensors are tethered to facilitate removal [11].

Functional challenges associated with fully-implantable devices include powering the implanted device and monitoring or changing settings on the device. Commercial pacemakers and defibrillators rely on non-rechargeable batteries, where the battery is the determining factor in the size of the implant, and periodic surgeries are necessary to replace the device due to the limited battery lifetime (every 5–8 years for pacemakers, every 3–5 years for ICDs) [1].

**Figure 1:**
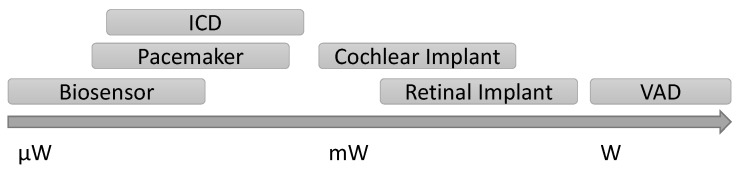
Range of power requirements of example implantable medical devices.

Depending on the application, implantable devices demand varying levels of power. For example, a pacemaker requires on the order of 10 μW–1 mW, while a retinal prosthesis requires approximately 45 mW, while a ventricular assist device (VAD) requires 5–25 W, as illustrated in Figure 1 [12,13,14,15]. Commercially available VADs still require percutaneous drive lines and an external battery pack due to their high power requirements [1,16]. There is also a considerable range of device sizes, with the VAD being much larger than a retinal prosthesis due to its functional requirements. A third consideration in addition to power level and size is whether a device needs continuous power. Interruption of power will impede the function of any device, but the consequences vary in severity. While interruption of power to a prosthesis will cause a decrease in quality of life and potentially secondary safety issues due to loss of sensory information, the case of a VAD power interruption is immediately life threatening. Meanwhile, an implanted biosensor may only need intermittent power to perform a sensor reading. These device considerations determine which powering methods are feasible for an implanted device.

### 1.1. Wireless Implantable Devices

Reviews and discussions of implantable device powering methods are provided in [14,17,18]. A focused comparison of inductive and ultrasonic energy transfer is provided in [19], and a review of acoustic energy transfer is given in [20]. Energy harvesting methods utilizing temperature gradients or piezoelectric materials have been developed, but as of yet cannot provide sufficient power for functioning implantable devices [15,17]. While the optimal powering method ultimately depends on the application, electromagnetic (EM) energy transfer has proven to be a promising wireless powering method with a broad range of system designs demonstrated in the literature, capable of delivering varying levels of power depending on size constraints of the device and the required function. Therefore, the focus of this review is on electromagnetic transcutaneous energy transfer.

Transcutaneous EM energy transfer has already enabled improvements to implantable devices by providing a method of powering an implantable device while reducing the dependence on implanted batteries and enabling remote communication with the implant. The cochlear implant was the first commercial wirelessly powered implantable device [7,8,21,22]. Wireless power is provided to the cochlear implant through several millimeters of tissue, along with communication of processed auditory information from an external microphone. Retinal prostheses are a recent development and have been successfully implanted in humans to restore sight [9]. There have also been research efforts towards a fully implantable wirelessly powered VAD, even with the challenging power requirements [16].

**Figure 2:**
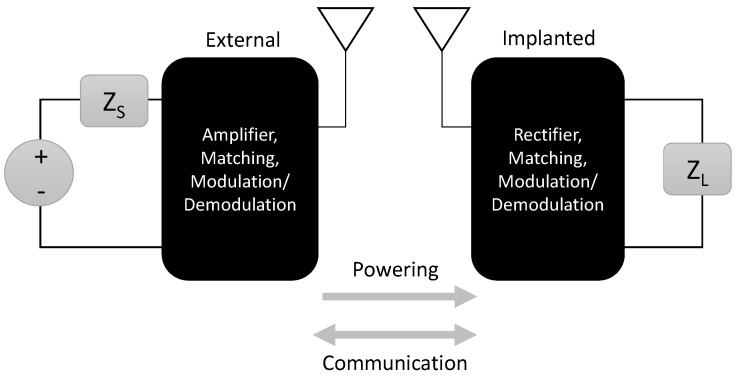
Simplified general wireless transcutaneous system architecture.

Wireless transcutaneous systems generally include an external antenna and an implanted antenna, as illustrated in Figure 2. EM energy is transmitted through the tissue from the external antenna, captured by the implanted antenna, and rectified to power implanted circuitry. The energy captured at the implant can be utilized to recharge a battery or to directly power a battery-less implanted device. Eliminating the implanted battery typically enables miniaturization of the implant, improving patient safety and comfort by lowering infection risk and lessening the obtrusiveness of the implant [1]. Depending on the power requirements and the need for continuous power, the power source of an implantable device can be primarily wireless, with a battery to provide backup power for critical functions, thereby prolonging the lifetime of the implant while providing a fail-safe for power interruptions [23].

Modulation of transcutaneous energy can be performed to achieve communication between the external and implanted sides. Back communication (implant to external) can be accomplished using a transmitter on the implant (active), or by modulating the energy from an external transmitter (passive), with active communication generally requiring more power at the implant. While the focus of this review is on electromagnetic energy transfer, similar communication has been achieved with other wireless powering methods such as ultrasound [24,25,26]. Wireless transcutaneous communication allows control of the implanted device behavior as well as access to information from the implant, such as readings from implanted sensors. This enables programmability of implanted devices and opportunities for post-surgical or remote long-term monitoring by reading information from the implant.

The main challenge associated with wireless transcutaneous energy transfer is achieving sufficient and reliable energy transfer to power the implant within safety limits on absorption in tissue. Electromagnetically, tissue is a lossy dielectric material with properties described by permittivity and conductivity. EM energy absorbed in tissue causes heating, hence there are fundamental safety concerns involved in transcutaneous energy transfer.

The goal of maximizing energy transfer to the implant while minimizing energy absorption in tissue has logically led to definitions of efficiency in terms of the power delivered to the implanted load, absorbed power, and input power [27,28,29]. The goal in the design of a system then becomes to maximize this efficiency. Conceptually, efficiency represents a distribution of input power in the source, antennas, system components, and load [30]. A more specific definition of efficiency for a particular system depends on multiple parameters, including: Antenna topologies, antenna dimensions and separation, the properties of the media surrounding the antennas, and operating frequency. These parameters determine the characteristics of the electromagnetic fields in the system. Once the system has been characterized, there are numerous strategies for optimizing the efficiency, whether through frequency tuning, impedance matching, or load tuning.

### 1.2. Environmental Variations

While a transcutaneous system can be optimized for a particular configuration, wireless transcutaneous energy transfer in practice is complicated by unpredictability and variability of the physiological environment. Variations occur due to antenna misalignment, movement of the antennas or the patient, implant migration, and changes in tissue structure and properties. Changes in the environment directly affect the performance and safety of a transcutaneous system, through effects on impedance and field characteristics. Impedance changes can reduce power transfer at the transmitter and receiver, as well as degrading efficiency if the impedance deviates from an optimal load or if there is increased absorption in tissue [31,32,33]. The degradation in power transfer and efficiency can lead to other problems in the system: Power reflections at the transmitter can damage the system, while reduced power delivery to the receiver can cause interruptions in function, and excess power delivery to the receiver could damage receiver circuitry or lead to tissue heating and safety concerns [34,35,36,37,38].

To avoid or lessen the degradation in system performance, sources of environmental variations must be examined and accommodated. One source of variation is changes in positioning or alignment of the antennas, whether due to movement of the patient or implant migration. Inductively coupled systems are particularly sensitive to variations in distance or alignment [39]. Some systems employ flexible antennas on the tissue surface, where movement can change the antenna geometry as well as the alignment [40]. In retinal prostheses, the implant antenna is in near constant movement due to its location in the eye [41]. Migration of an implant can occur after implantation, resulting in uncertainty of the implant location and potential misalignment of the antennas [42]. Distance or alignment variations can cause reduced power delivery and interruption of the implant function [43].

Although less attention is devoted to potential changes due to differences in tissue characteristics, there is evidence that tissue varies among patients and over time [44,45,46,47,48,49,50,51]. These variations are in addition to the known frequency dependence of tissue dielectric properties. Changes in body chemistry (such as hydration or fat content), inflammation, fibrous encapsulation, and changes in cellular structure are well-documented physiological processes that affect tissue properties. Changes in tissue thickness will result in variable separation between an external and implanted antenna, causing either increased coupling due to thinner tissue or greater attenuation of the fields through thicker tissue. Changes in tissue structure or tissue chemistry, and associated changes in tissue properties, will affect the antenna electrical size and impedance as well as the optimal frequency of operation.

Attempts to minimize the effects of variations have been realized through design of insensitive systems and adaptive systems. Insensitive systems are designed for consistent operation over a range of environmental parameters, without the use of tuning components [35,52]. Adaptive systems include tunable components to adjust the characteristics of the system in response to changes in the environment. The focus of this review is adaptive systems specifically designed for transcutaneous operation in the electromagnetic near or mid-field.

### 1.3. Review Organization

There is a wealth of literature on wireless power transfer, energy harvesting, antenna design, and tissue electromagnetics. Design concepts and challenges from each of these fields are combined in the design of wireless implantable medical devices. As such, there is much that cannot practically be covered in the background for this review. Although there are significant biomaterials and biocompatibility challenges associated with implantable medical devices, the focus of this work is on electromagnetic wireless transcutaneous powering of implantable devices. Additionally, the literature on electromagnetic wireless power transfer covers many components of system design, including power amplifier design, rectifier design, antenna design and voltage regulation. These components are essential and have been studied in terms of improving efficiency, and background information is provided in this work where relevant. Similarly, this work will address but not focus on aspects of wireless communication in transcutaneous operation, instead maintaining the focus on adaptive methods for transcutaneous wireless power transfer. The background of this review is meant to provide a general overview of concepts relevant to transcutaneous power transfer and the adaptive methods covered later in the paper, consolidating information from the many fields of research that contribute to transcutaneous powering of implantable devices.

The paper is organized as follows: Section 2 covers background topics relevant to the review of the literature on adaptive transcutaneous systems; Section 3 provides an overview of the design process for an adaptive system and the strategies that have been implemented or proposed for each stage of the process; Section 4 details implementations in the literature specifically for implantable devices and transcutaneous power transfer; finally, Section 5 provides a summary of the literature and a discussion of remaining challenges in the field.

## 2. Background

### 2.1. Wireless System Architecture

A wireless system typically consists of the components shown in Figure 2. At the primary or transmitter (external), the ac transmit signal is generated and fed to the transmit antenna. The EM field generated by the transmit antenna is captured by the receive antenna and converted to a dc voltage to power the receiver circuitry.

Oscillators and power amplifiers are common at the transmitter to generate the ac transmit signal. The power amplifier class and amplifier efficiency are design concerns at the transmitter. A matching network is often included to match the transmit antenna to the optimal load of the power amplifier to improve efficiency. At the receiver, rectifier efficiency is a design concern, as well as voltage regulation to supply a stable voltage to the receiver circuitry. The choice of transmit and antenna topology and dimensions depends on the application.

### 2.2. Field Regions

Wireless systems can operate in various field regions, depending on the operating frequency, antenna size, antenna separation and transmission medium. Field regions are characterized by the types of fields present. The reactive near field is closest to the radiating antenna and contains primarily “stored” energy; the radiating near field contains both radiating and reactive fields with radiating fields dominating; the far field contains radiating electric and magnetic fields in planes transverse to the direction of propagation and the angular field distribution can be considered independent of distance from the radiating antenna [53,54].

Definitions of the boundaries between the fields vary and the transition between regions is gradual, but the region boundaries can be approximated in terms of the electrical size of the antennas and the electrical distance between them (relative to the wavelength at the operating frequency) [53,54]. The electromagnetic field region determines the interaction (if any) of the transmitting and receiving antennas, which guides the associated design and analysis of the system. Theoretically, any antenna system can be made to operate in any field region by varying the size or separation of the antennas. However, the efficiency of a system is expected to degrade when operating outside of the desired field region to the point where the system may cease to function.

Systems designed to operate in the reactive near field include inductively coupled or magnetically coupled systems, where the magnetic field of a transmitter coil induces a current in a receiver coil [40]. The term “antenna” is not typically used to refer to the coils in inductively coupled systems. Rather, the term “coil” or “resonator” is used to designate that the coils are designed as coupled (reactive) and not as radiating antennas. Capacitive coupling via the electric field is also possible in the reactive near field, and has enabled power transfer to implantable devices in proximity to metallic implants [55]. Efficiency in an inductively coupled system decreases significantly with greater coil separation or disparate transmit and receive coil dimensions due to reduced coupling [12,29,42]. This effect tends to limit the miniaturization of implantable inductively coupled devices. Magnetic resonance systems have been shown to have high efficiency up to antenna separations of 1–2 coil diameters by maximizing the coil quality factor, but efficiency is still dependent on similar size of the transmit and receive resonators [12,29,56].

Weakly coupled systems can be designed to have higher efficiency in the radiating near field region by deliberately utilizing differently sized antennas [57]. Such a system can be designed with the receive antenna dramatically miniaturized relative to the transmit antenna. This “midfield” region of operation has been recently investigated for transcutaneous powering of miniature implantable devices.

Far field systems operate at a distance and frequency such that there is no detectable coupling between the transmit and receive antennas [54]. This region is less used for implantable devices to avoid problems with attenuation of propagating fields in tissue. The focus of this review is on transcutaneous systems with an external antenna on or near the tissue surface and an implanted antenna, limiting the discussion to reactive near field and midfield systems.

For transcutaneous applications, inductively or magnetically coupled systems are well suited when the coil separation can be on the order of the coil diameter to maintain high efficiency, and where the external and implanted coils can be of similar size. Such systems have been shown as capable of delivering tens of watts of power to an implanted device at small coil separations [12,43]. In cases where miniaturization of the implanted device is a priority, the separation between the implanted and external antennas is likely to be larger than the antenna dimensions. Therefore, the efficiency will be maximized by designing for midfield operation with asymmetrically sized antennas. Such systems have been shown to achieve power delivery of up to several milliwatts to a millimeter-sized implant [15].

### 2.3. Tissue Properties

Two main current paths can be identified in tissue, corresponding to permittivity and conductivity and the current paths described in Ampere’s Law. One path is conduction current, corresponding to the movement of ions in tissue and therefore the conductivity (*σ*). The other path is displacement current, a function of the electric flux and permittivity (*ϵ*).

Conductivity is a function of ionic mobility in the tissue medium, where ions such as sodium and potassium act as charge carriers. Permittivity is a function of charge buildup at cell membranes (cell membrane capacitance) and alignment of molecular dipoles with an applied field [58]. Dielectric relaxation is also included in the complex permittivity, describing the effect of molecular dipole rotation delay in response to an applied field. Tissue conductivity and permittivity are frequency dependent, with conductivity increasing with frequency, and permittivity decreasing with frequency.

In general, conduction current can be utilized at low frequencies and small antenna (electrode) separations, where the movement of ions creates capacitive charge transfer. At higher frequencies or greater antenna separations, conductivity contributes to loss due to field vector directions and the inability of the ions to build up along interfaces. At higher frequencies, displacement current becomes significant, and the induced current due to greater flux competes against conductive losses. At GHz frequencies, dielectric relaxation losses also become significant. The specifics of energy transfer differ based on the tissue structure, thickness, and chemistry. Tissue properties have been measured for multiple tissue types across frequency, and parametric models have been developed to represent tissue properties over a range of frequencies [47,58].

Tissue properties are particularly relevant to wireless power transfer due to their relationship to power dissipation in tissue, which can lead to tissue heating and therefore presents safety issues. Energy absorption in tissue is quantified as specific absorption rate (SAR), measured in watts per kilogram. SAR limits defined by several organizations are used as safety guidelines for transcutaneous energy transfer. These limits have been defined based on studies of physical and behavioral effects of electromagnetic field exposure in animals and humans, but are not specific to medical use.

The IEEE Standard for Safety Levels with Respect to Human Exposure to Radio Frequency Electromagnetic Fields 3 kHz–300 GHz (IEEE Std C95.1-2005) specifies limits of 0.4 W/kg and 10 W/kg for whole body and local SAR, respectively [59]. The International Commission on Non-ionizing Radiation Protection (ICNIRP) guidelines specify that general public exposure is limited to 0.08 W/kg whole body, 2 W/kg local for head and trunk, and 4 W/kg local for limbs, with local SAR averaged over 10 g of tissue for any 6 min period [60]. For uncontrolled exposures of the general population, the U.S. Federal Communications Commission (FCC) limits SAR to 1.6 W/kg averaged over any 1 g cube of tissue [61]. In addition to dielectric losses, heating can also occur due to heat dissipation of the receiver circuitry, sometimes exceeding the one-degree Celsius temperature increase that is a basis for SAR limits [41,62,63].

### 2.4. Operating Frequency

Due to the complex frequency dependent dielectric properties of tissue, the existence of an optimal frequency has been proposed that balances power transfer and losses in tissue [27,28]. The optimal frequency was determined by defining a measure of efficiency in terms of power delivered to the load and power absorbed in tissue [27]. Ho *et al.* [57] showed that the highest efficiency is achievable in the midfield for weakly-coupled disparate-sized antennas, resulting in an optimal frequency in the sub-GHz to GHz range for a cm-size transmitter and mm-size receiver at cm-separation. Higher frequency has also been recently investigated as it enables miniaturization of antennas, greater antenna impedance, and greater open circuit voltage at the implant [28,32,39,57,64].

### 2.5. Transcutaneous Antennas

Particular antenna topologies tend to be favored for transcutaneous systems due to size constraints and the effects of tissue properties. Miniature antennas are desirable for implants to reduce patient discomfort and infection risk, but this results in electrically small antennas where impedance mismatch can lower efficiency [65]. Miniature antennas have been shown to be most efficient in weakly coupled systems operating in the midfield [27].

Tissue’s high permittivity (due to its composition of mostly water) reduces the net electric field, an effect of molecular alignment with an applied field. Loop or coil antenna topologies are widely used in the implantable device literature to take advantage of tissue’s non-magnetic permeability and utilize the magnetic near field. Fewer turns have been shown to be better in proximity to conductive high permittivity tissue, with quality factor decreasing with increasing turns [28]. The induced current at a loop receiver depends on the flux through the loop, hence the advantage of using higher frequencies. At frequencies and antenna dimensions such that there is midfield operation, it has been shown that both the electric and magnetic fields contribute to power transfer [66].

Inductive antennas (loops) act as better receivers due to their low impedance, while capacitive antennas act as better transmitters due to their larger impedance which limits current flow at the transmitter [14]. Low impedance can lead to power loss and heat generation due to high currents when used as a transmitter [39].

Printed antennas are favored due to the potential for miniaturization and manufacturability [28]. More complex antenna designs have been investigated to focus fields at the site of the implanted receiver, and to minimize excess power dissipated in tissue [15,42,67].

### 2.6. Power Gain and Efficiency

Because the function of any receiving device depends on its power supply, it is desirable to maximize power transfer to an implant. However, transcutaneous operation necessitates consideration of safety limitations relating to absorption of electromagnetic energy in tissue. The need to maximize power to the load while minimizing absorption in tissue then evokes definitions of efficiency.

Definitions of efficiency vary in terms of what the load power represents and what it is expressed relative to: power available to the load relative to the power absorbed in tissue [15,27,42]; power to the load relative to power available from the source, representing the transducer gain [68]; power to the load relative to power delivered by the source, with maximum efficiency achieved with an optimal load [32]; power to the load relative to power input to the network, maximized with a conjugately matched load in a weakly coupled system [57,66]. Optimal load impedances have been derived to maximize efficiency according to the various definitions [29,32,57]. The definitions of efficiency overlap with definitions of power gain, but for passive systems the term efficiency is used to indicate that the power gain from source to load is less than one.

Defining efficiency relative to the power from the source, there exists an upper bound of 50% power efficiency with simultaneous conjugate matching [32]. While the load resistance is equal to the source resistance for maximum power transfer, the load resistance is larger than the source for higher efficiency (relative to the source power) [29]. Designing for maximum efficiency in this case may reduce the range of operation due to reduced power transfer to the load [29]. However, maximizing voltage at the implant is sometimes preferable even at the expense of power transfer, to ensure adequate turn-on voltage while minimizing losses in the system. Additionally, in the case of delivering power from a power amplifier, efficiency (defined as output power from the power amplifier relative to input power) is prioritized to minimize heat dissipation in the circuit. In this case, the power amplifier is designed to operate at maximum efficiency, rather than using conjugate matching, and an optimum load can be defined [32,69].

Defining efficiency as load power relative to power absorbed in tissue, maximum efficiency is achieved through conjugate matching [27,57]. For delivering power to a load through tissue, this efficiency is valuable when power dissipation in tissue is of more significant interest than power dissipation elsewhere in the system.

The system efficiency or gain can be decomposed into component efficiencies, including coupling efficiency that represents the efficiency between the transmit and receive antennas (coils) [70], power amplifier efficiency [32], and rectifier efficiency [71,72,73]. In midfield cases where losses in tissue are significant and lumped element models are no longer appropriate, network analysis allows representation of a linear system in terms of scattering parameters (S-parameters) to calculate efficiency or power gain [27,69,71,74].

### 2.7. Impedance Matching

Impedance matching networks can be added to transform the real and reactive impedances looking into portions of a system, to minimize voltage reflections at interfaces, to conjugately match for maximum power transfer, or to achieve optimum loading.

Common network topologies include L, T, and pi networks consisting of inductors and capacitors in configurations capable of increasing or decreasing input impedance. The choice of topology depends on design parameters including the desired bandwidth, impedance transformation range, complexity, and available area [75].

Pi-match networks can be used to both increase and decrease impedance, while L-match networks can only be designed to transform the impedance in one direction (either increase or decrease) [69]. Pi networks can provide wider band matching, but L-match is appropriate when efficiency is of primary concern [32].

### 2.8. Impedance and Material Properties

Impedance is closely related to material properties of conductivity, permittivity, and permeability, and properties of inductance, capacitance, and resistance. Impedance is typically represented as a function of resistance (*R*) and reactance (*X*) as given in Equation (1). Resistance is then a function of geometry and conductivity, while reactance is a function of geometry and permittivity or permeability. Reactance can also be expressed in terms of inductance (*L*) or capacitance (*C*), also indicated in Equation (1).

(1) $$Z=R+jX=R-\frac{j}{\omega C}=R+j\omega L$$

### 2.9. Resonance

Resonance in the context of antenna or circuit design typically refers to a system with completely real input impedance. A natural resonance associated with the system geometry has also been defined as where the determinant of the scattering matrix approaches zero [65]. For the purposes of this review, the focus will be on the first definition of resonance due to its relevance to impedance matching. Resonance tuning is equivalent to impedance matching to achieve an all-real input impedance, implemented to maximize power delivered to or from a real impedance. Designing antennas with inherent resonance can avoid losses associated with an added matching network. Operating transmit and receive antennas at the same resonant frequency increases the voltage gain between the antennas [13,41].

Near field systems tend to utilize resonant tuning to design transmitting and receiving antennas (coils) to function at the desired operating frequency. Resonance tuning can include added capacitors or inductors to compensate for an antenna’s reactive impedance, or antennas designed such that the inherent capacitance and inductance are tuned to resonance [65]. Magnetic resonance systems have been designed with three or four coils, where the additional coils perform impedance transformation at the transmitter and/or receiver [56,76]. This is assuming that the coils are being designed with a specific frequency in mind. In some cases, the frequency is instead tuned to achieve resonance with the given impedance characteristics of the system. It has been noted that the optimum load differs from resonant tuning if the media between the transmit and receive coils is conductive, as in the case of transcutaneous operation [32].

### 2.10. Quality Factor

Quality factor (*Q*) is a measure of the energy stored in a system relative to the energy dissipated or lost per time. For a passive parallel RLC network at resonance, *Q* is defined as in Equation (2) [69]. The stored energy ($E_{stored}$) is the peak energy stored in the capacitance (*C*) or inductance (*L*), as the oscillatory energy is transferred between the two. The power dissipated ($P_{avg}$) is the power through the resistance (*R*).

(2) $$Q=\omega_{0}\frac{E_{stored}}{P_{avg}}=\frac{R}{\sqrt{LC}}$$

*Q* represents the ratio of the current flowing in inductors/capacitors to the net current through the network. Therefore, operating at high *Q* increases the voltage swing across the LC part of the network. Higher *Q* also corresponds to narrower fractional bandwidth, as shown in Equation (3) [69].

(3) $$\frac{BW}{\omega_{0}}=\frac{1}{Q}$$

High *Q* for an antenna equates to low radiation efficiency, and there is a fundamental limit on the minimum *Q* of electrically small antennas [54]. One strategy for achieving better radiation properties is space-filling or meandering, where an antenna occupies an equivalent area but is made to have an increased electrical length [54]. In the case of near field operation, however, designing electrically small antennas to operate at high *Q* is desirable to increase the “stored” near field energy. The basis for magnetic resonance systems is designing coil antennas to operate at high *Q* [12]. Because high *Q* equates to narrower bandwidth, coupled systems can be more efficiently designed using separate coils (and separate frequencies) for power and data [73].

*Q* of a material is inversely related to the loss tangent (tanδ) of the material, defined in Equation (4) in terms of conductivity and permittivity.

(4) $$\tan\delta =\frac{1}{Q}=\frac{\sigma }{\omega \epsilon }$$

## 3. Components of Adaptive Transcutaneous Systems

The goal of adaptation in any system is to maintain desired performance despite environmental variations. How “performance” is defined and the strategies to maintain performance depend on the application and the operating field region of the antennas.

**Figure 3:**
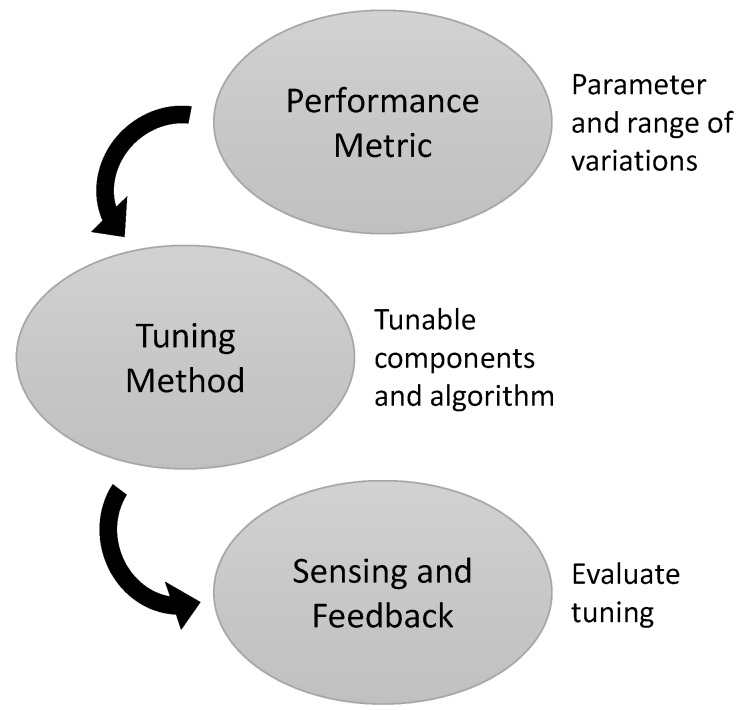
General process steps in designing an adaptive system.

The general steps in the design of an adaptive system are illustrated in Figure 3: (a) Defining a parameter to optimize (a performance metric) based on the functional goal of adaptation; (b) developing a tuning method based on the available controls affecting the performance metric and the expected range of parameter variations; and (c) implementing sensing to determine or evaluate the tuning state. This section will provide a conceptual overview of the strategies employed in each stage of the design, and the following section will cover specific implementations in the literature.

It should be noted that the focus of this review is on adaptive methods for electromagnetic wireless transcutaneous systems. There is a substantial amount of literature on adaptive electromagnetic wireless systems that is outside the scope of the current review, but methods from these areas are included where they have been applied to transcutaneous power transfer to implantable devices.

### 3.1. Performance Metric

The parameter used as an indication of system performance depends on the desired function of the system and the system characteristics. Strategies include minimizing reflections at interfaces, maximizing power transfer, maximizing efficiency, and maintaining constant load voltage.

Maximum power transfer can be maintained in variable environments through tunable impedance matching. Minimizing reflection achieves maximum power transfer when the goal is matching to an all-real impedance. When matching to complex impedances, maximum power transfer is achieved through complex conjugate matching.

There are various definitions of efficiency, but power efficiency is typically defined as load power relative to input power or power absorbed in tissue [27,32]. Design strategies to maintain efficiency in the presence of environmental changes are many and varied, but most can be generally classified into frequency tuning and impedance tuning.

### 3.2. Tunable Components

Tunable components are essential to adapting a system with the goal of optimizing a chosen parameter. In frequency tuning applications, a voltage controlled oscillator can be used to adjust the transmitter frequency [40,77,78]. The switching frequency of the power amplifier at the transmitter can also be controlled [73].

In impedance tuning applications, variable inductances and capacitances can be used in matching networks to transform impedances. Transductors provide current-controlled inductance, and can withstand large voltage and current, but they tend to be too bulky for wearable or implantable devices [40,79]. Microelectromechanical systems (MEMS) inductors have been shown to provide high efficiency and tuning range, but variable inductors remain difficult to implement on-chip for miniature implants [80,81]. Variable capacitances tend to be favored over inductances due to the widespread use of inductive coil antennas and the higher quality factor of capacitors on-chip [32,75,78]. Voltage-controlled capacitors (varactors) offer compact area, but they can exhibit non-linearities at radio frequencies and require an analog control voltage [40,64,68,75,82]. Switched capacitor banks have also been used to vary parallel matching capacitance, but their disadvantages include the greater space occupied and the necessary discretization of the capacitance values [76,83,84,85].

Another approach to impedance tuning is duty cycling. Ahn *et al.* [76] implemented buck-boost converter load connection duty cycling to modulate effective load in an inductively coupled system; Si *et al.* [83] duty-cycled switched capacitors to achieve variable effective capacitance. Variable output dc-dc converters and control of power amplifier supply voltage have been used to regulate supply voltage and input power at the transmitter [37,40]. In some cases the antenna itself can become a tunable component, either through adjusting multiple feed points to achieve beamforming, or through changes in the antenna geometry [15,86,87].

### 3.3. Sensing and Feedback

**Figure 4:**
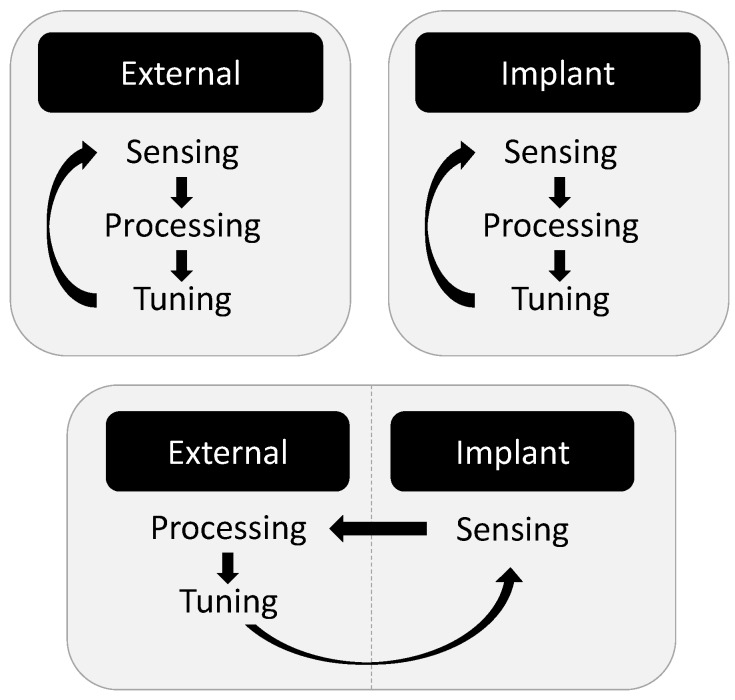
Existing approaches to adaptive transcutaneous system design.

Feedback can be implemented to evaluate the state of the performance metric and to determine the appropriate control of tuning components. The various approaches in the literature fall into three categories, illustrated in Figure 4: (1) Sensing and processing at the external transmitter to tune components at the transmitter; (2) sensing and processing at the implanted receiver to tune components at the receiver; and (3) sensing at the implanted receiver communicated to the external transmitter for processing and tuning components at the transmitter.

Parameters used for feedback include the rectified voltage indicating received power [40,88], reflected voltage indicating impedance mismatch [77], and phase differences to detect reactive impedance [40]. Information on parameters sensed at the receiver can be communicated to the transmitter passively by modulation of the power carrier, or actively through the use of an implanted transmitter.

The tuned components, sensed parameter, and the control method are closely related. The control method is designed based on the parameters available to sense, the controllable parameters that affect the optimized parameter and the subsequent choice of tunable components, and any design constraints on tuning time, size, and power consumption.

The tuning time must be controlled such that it does not interfere with primary functions of the system such as communication. An iterative algorithm such as gradient search is relatively simple to implement, but has a disadvantage in tuning time compared to single iteration methods based on direct calculation [80]. However, the power and memory requirements of complex vector calculations can limit the application of such methods on miniature implantable devices. A balance must be achieved among tuning time, complexity, and accuracy.

Adaptation control can be implemented at either the transmitter or receiver, or both the transmitter and receiver. Strategies at the transmitter include adjusting transmitter power and frequency, or tunable matching at the transmit antenna [12,34,36,37,40,43,82,83,89,90]. Adaptation strategies at the receiver mainly include tuning matching networks to achieve optimal load [72,90,91,92].

Adaptation of both sides of the network is necessary for maximum power transfer efficiency, and may require communication of the adaptation state from one side of the network to the other [64,70]. For any adaptation strategy to extend to an implantable device, it must be miniature and low power to avoid adding significantly to the existing power and size requirements of the implant. There are fewer restrictions on the transmitter in terms of size and power consumption, but safety is still a primary concern when transmitting power through tissue [14].

## 4. Implementations of Adaptive Transcutaneous Systems

The combination of strategies discussed above depends on which environmental variation(s) are addressed and where in the system the adaptation is performed. A review of implementations in the literature is given in Table 1, indicating the goal of adaptation (including the performance metric), tuning performed and feedback at the external transmitter (Tx) and/or the implanted receiver (Rx), and the environmental variation for which the adaptation is intended to compensate. Designs of interest are categorized and discussed in the following subsections.

### 4.1. Input Power Adjustment

Changes in the separation distance and alignment of antennas can cause variations in coupling and power transfer [95]. One strategy to compensate involves regulating input power at the transmitter. The motivation for this tuning is to avoid or reduce the power regulation burden at the implant, which contributes to heat dissipation [39].

Van Schuylenbergh and Puers [40] adjusted the transmit power using a voltage-controlled boost regulator, based on feedback of the dc input voltage at the internal regulator.

Wang *et al.* [36] compensated for coil movement or load changes, regulating transmitted power by adjusting the supply voltage to a power amplifier. The supply voltage was adjusted according to the detected voltage on a storage capacitor at the receiver, communicated to the transmitter over a second frequency band. Power level adjustment and back telemetry were staggered to avoid interference.

Si *et al.* [39] demonstrated power regulation at the transmitter, designed to limit the size and heat dissipation associated with power regulation at the receiver. Power was regulated by adjusting the supply voltage at the primary resonant converter based on feedback on the dc voltage at the receiver, communicated wirelessly to the primary.

Ng *et al.* [14,41] proposed a system to compensate for eye movements and fibrous growth in retinal implants, regulating the supply voltage at the transmitter based on feedback on the voltage at the secondary, communicated through back telemetry. Adjusting the transmitter resonant frequency for maximum efficiency by tuning the transmitter capacitance was also proposed, based on voltage at the receiver. A primarily theoretical discussion was presented for tuning and feedback.

Kiani and Ghovanloo [37] designed a system to compensate for distance and angular alignment variations in an inductively coupled system, regulating transmit power based on an indication of voltage across the receive coil. Back telemetry was performed if the received voltage was greater than a reference value, so the transmitter increased transmit power unless bits were received from the implant. The design focused on using primarily off-the-shelf components.

Waters *et al.* [23] designed a system to adjust transmit power based on reflected voltage at the transmitter to compensate for coupling variations in a magnetic resonance system. They later proposed an auto-tuning algorithm to adjust transmit power based on detected load power, and measured the temperature of the receive coil as a measure of efficiency [12]. Back telemetry of load power was mentioned as future work.

### 4.2. Adaptive Antennas

In an effort to compensate for changes in alignment of the antennas, which can lead to changes in coupling and power transfer, transmitters have been designed with location and focusing capabilities. The concept is related to power regulation, but the regulation can involve multiple antennas or control of multiple feeds to a single antenna.

McMenamin *et al.* [96] presented a system that adjusted powering of antennas in animal cages for bio-telemetry experiments based on the animal’s detected position. The goal was to focus wireless electromagnetic energy to power a mobile telemetry unit worn by the animal. The floor of the animal cage consisted of an array of overlapping planar spiral coils, and the magnetic field within the cage was focused by only providing power to the coils closest to the detected position of the mobile telemetry unit. A small magnetic tracer was embedded in the mobile telemetry unit and an array of magnetic field sensors in the cage floor was used to detect the unit’s location.

Ho *et al.* [15] manipulated the field pattern itself to maximize efficiency by implementing a transmitter with dynamic focusing capability. The transmitter was designed to power a miniature (2 mm × 3.5 mm) implant in the midfield (5 cm separation) for pacemaker or cortical implant applications. Through control of the phases of the antenna feeds, the field was focused at an implant in various locations. The focusing was adjusted using an optical indication of received power as feedback for the purpose of the experiment. A method of back telemetry to practically adjust the focusing was not discussed.

### 4.3. Frequency Tuning

Frequency tuning has been utilized to correct for changes in antenna impedance due to the surrounding environment, including maintaining operation at a resonance frequency. Frequency tuning can be difficult due to FCC regulations on frequency bands [84,91]. It is mainly used in inductively coupled systems, where the coils can be designed such that the coupled system does not radiate significantly, but the operation is highly affected by coil separation distance [68]. A review of automatic frequency control techniques is provided in [97], and several examples of frequency tuning in transcutaneous systems are presented here.

Ko *et al.* [77] used a voltage-controlled oscillator to adjust the transmit frequency in response to detuning of an animal’s cage for bio-telemetry experiments. The goal was to maintain operation at the resonant frequency of the transmitter circuit including the animal’s cage, in order to power a battery-less implant for chronic animal telemetry. Detuning due to the animal’s movement was detected by measuring reflected voltage at the transmitter, and the transmitter frequency was adjusted to minimize reflections. Phase-lock loop techniques were proposed as a strategy for tuning the receiving unit in response to changes in the transmitter frequency.

Fernald *et al.* [78] also used a voltage-controlled oscillator, and adjusted the transmit frequency in response to changes in the antenna resonant frequency. The application was a general-purpose implant for animal telemetry experiments. The system performed a resonant search, sweeping the frequency and monitoring the voltage amplitude across the transmit antenna. When the voltage swing reached a threshold value, the frequency was fixed and the system began transmitting at that frequency.

Baker and Sarpeshkar [73] presented a class-E controller to compensate for changes in coupling, comparing the transmit resonator voltage to a reference voltage and controlling the amplifier switching, with the goal of maintaining link efficiency with robustness to changes in coil separation. They also present a feedback control analysis of a coupled system for wireless electromagnetic power, and an experimental investigation of the effects of coil separation on peak efficiency. Experimental tests of their switching control system showed less than 16% variation in rectified output voltage over coil separations of 1–10 mm.

Ahn and Hong [34] adjusted operating frequency to maintain constant output voltage with coupling and load variations. The goal was to implement a low-power solution without requiring complex active circuits or external components. The switching frequency of a self-oscillating class-D power amplifier was controlled based on feedback from the drain of the switch transistor. The frequency-tuning system was demonstrated to have relatively constant output voltage over load variations and distances up to 12 mm, and able to maintain constant voltage over greater distances at higher load resistance.

Wang *et al.* [43] implemented a zero voltage switching follower design to compensate for coupling variations in inductively coupled systems, with the goal of providing power to an implantable heart pump. Switching frequency control was performed based on voltage feedback at the transmitter, in order to maintain middle zero voltage switching operation. The output power and efficiency were measured with and without frequency control, and the tuned system was able to deliver 10 W at greater coil separation and higher efficiency than without frequency control.

### 4.4. Impedance Tuning

Frequency tuning has been paired with automatic impedance matching to achieve resonance of an antenna while maintaining matching to a feed line. Van Schuylenbergh and Puers [40] adjusted the transmit frequency using a voltage controlled oscillator based on feedback of the dc input voltage of the internal regulator, communicated from the receiver using a third external sensing coil. Variable impedance matching at the transmit coil was also implemented based on the tuned transmit frequency, using a phase comparator to detect detuning of the coil. Hirata *et al.* [82] used two varactor diodes to tune the resonance frequency of a coil and to match the coil impedance to a transmission line. The matching was evaluated by sensing the phase change of reflected voltage at the transmitter.

**Figure 5:**
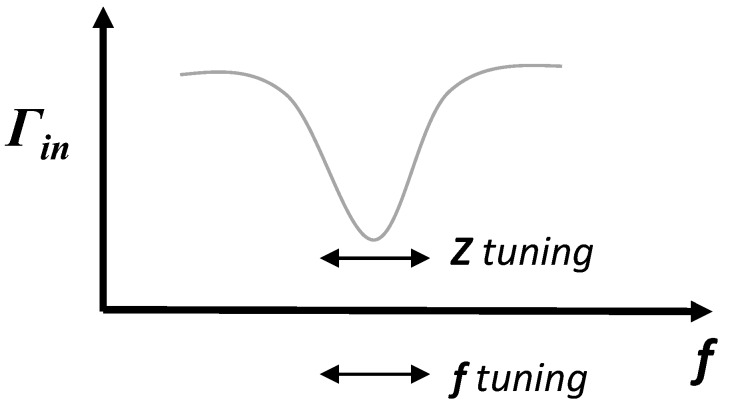
Illustrated relationship between impedance (*Z*) matching and frequency (*f*) tuning.

Automatic impedance matching has also been implemented in systems operating at a fixed frequency to compensate for impedance variations that detune the system and decrease power transfer and efficiency. Impedance mismatches can be the result of antenna position or impedance changes due to tissue parameter variations or movement. It has been suggested that although frequency tuning is easier to accomplish, higher efficiencies are possible with impedance matching [68]. Adaptive matching and frequency tuning are two methods of adjusting the system to operate at a desired matching state; one adjusts the impedance of the system to achieve matching at a fixed operating frequency, while the other adjusts the frequency to operate at a matched system impedance. This is illustrated in Figure 5, with matching indicated as minimizing the reflection coefficient ($\Gamma_{in}$).

Si *et al.* [13,83] implemented switched capacitors to control effective capacitance in a push-pull resonant converter to achieve resonance at a given reference frequency, to compensate for changes due to load or circuit parameters. Rodes *et al.* [94] developed a tuning system for adjusting capacitance of a half-bridge voltage-mode resonant converter. The work referenced [13,83], stating that the half-bridge voltage mode resonant converter is better suited to medical applications due to the current-fed push-pull converter’s inductances and the error terms in the switched capacitor transfer functions.

Waters *et al.* [84] demonstrated automatic impedance matching to match transmit and receive coil resonator input impedances to the source and load impedances, to accommodate changes in coupling of a magnetic resonant system due to changes in distance between the resonators. The demonstrated systems use extracted S-parameters to calculate the states of tunable matching networks. In the first case, the ideal matching impedance is calculated from the S-parameter matrix; in the second case, the matching state is found using an optimization algorithm, calculating the system efficiency from the S-parameters. The use of a network analyzer to extract S-parameters precludes direct application of this method in a real-time implantable system.

Chan Wai Po *et al.* developed a method of calculating complex antenna impedance based on detected voltage on capacitors, and using the calculated impedance to determine and set tunable reactive components in a matching network [31,80,89,93]. Implementations included MEMS variable inductors, switched capacitor networks, and varactors. The system was designed to compensate for impedance changes in pacemaker applications due to tissue differences, patient position, or nearby objects. Their one-iteration method reduces the time required for tuning, but requires vector calculations. Tuning power requirements are not provided, but it is proposed that the method reduces power requirements due to reduced tuning time. The method is applicable to both the transmitter and receiver, stated as the first system able to match both in a single process [31]. The transmit antenna impedance is matched to a power amplifier, while the receive antenna impedance is matched to a low noise amplifier.

Park and Ghovanloo [92] controlled for impedance variations due to the environment surrounding an intraoral sensing device. Switches in a CLC pi matching network were controlled by a microcontroller and set according to the output power monitored via a power detector. The system swept 16 possible settings and monitored output power, then set the configuration to the optimal switch setting, in a process taking 30 ms and repeated every second. However, the tuning was acknowledged to potentially impede real time operation due to interrupting communication of the intraoral device to an external receiver. The application was not specifically an implantable device, but was designed with size constraints to be worn on a dental retainer and addressed many of the same issues of energy transfer through tissue.

O’Driscoll *et al.* investigated the effects of misalignment, implantation depth, and tissue composition on system impedances in an implantable device, with focus on devices for neural recording [64,71,81,90]. The impedance was adjusted using a switched capacitor array, based on feedback of the voltage across the resistive load. The algorithm was a hybrid of gradient and binary search, where only the sign of the gradient was calculated to save power. The capacitor array and control were implemented on-chip, with the adaptation algorithm off-chip. The method is applicable to matching at both the transmitter and receiver.

Carta *et al.* [79] designed a self-tuning inductively coupled system to compensate for misalignments and distance changes, using a switched capacitor bank at the transmitter. The capacitor values were switched based on the voltage at the receiver, in a two stage process. First, the capacitance combinations were varied to determine the highest induced voltage at the receiver. Then, the capacitance was adjusted to maintain the voltage level determined in the first step.

Zargham and Gulak [72] developed a system for maximizing efficiency by tuning the load impedance to an optimal load. A switched capacitor array was controlled according to a sign-based gradient descent algorithm to maximize the power delivered to the rectifier load at the receiver. The implant receiver coil and circuitry were implemented on a single die in CMOS.

## 5. Summary and Future Directions

Miniature implantable devices are increasingly desirable to lessen the intrusiveness of the device for the patient and to reduce surgical complexity and infection risk. Electromagnetic energy transfer enables transcutaneous powering and communication with fully-implantable wireless medical devices, lessening the dependence on an implanted battery. The primary goal of transcutaneous energy transfer is to provide sufficient power to the implanted device while minimizing tissue heating due to absorbed energy. This has led to extensive research toward maximizing efficiency, through optimizing operating frequency, impedance matching and power delivery.

A significant complication of transcutaneous energy transfer is the variability of biological tissue in terms of tissue structure and electromagnetic properties. Tissue thickness and properties vary among individuals, among areas on the body and over time. Therefore, optimizing a transcutaneous system for a particular environment does not guarantee consistent operation in a real application.

**Figure 6:**
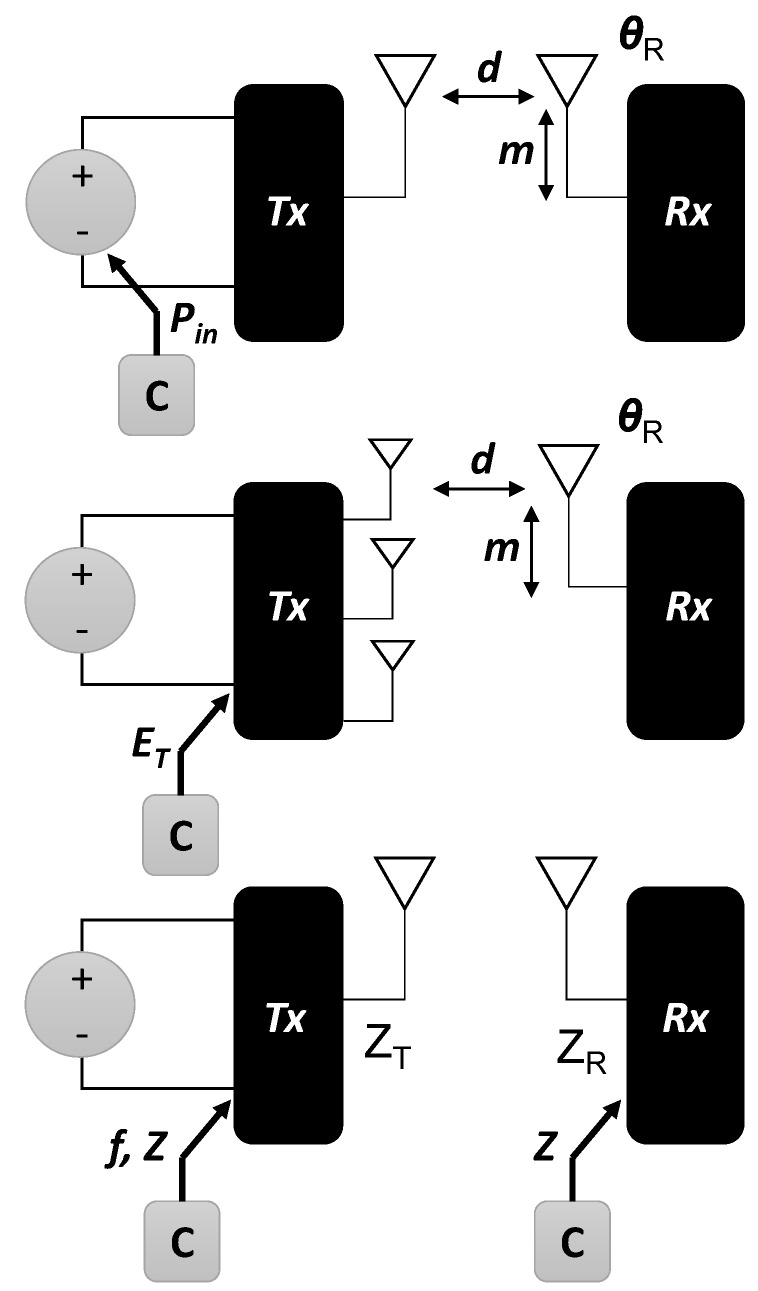
A graphical summary of tuning methods in the literature.

Recent research has included adaptive functionality to compensate for variations encountered in the practical use of a transcutaneous system. These adaptive methods include tuning components of the system to maximize power to the implant within safety limits on absorption in tissue. Strategies at the external transmitter include power regulation, frequency tuning, beamforming and impedance matching to improve power transfer. Strategies at the receiver primarily involve impedance matching. Feedback provides closed loop control, including sensing at the receiver and communication to guide adjustments at the transmitter of power or frequency. A graphical summary of adaptive implementations in the literature is provided in Figure 6, including: input power ($P_{in}$) adjustment to compensate for changes in antenna distance (*d*) and alignment (*m*, $\theta_{R}$); transmit antenna feed (field pattern $E_{T}$) adjustment to compensate for changes in implant antenna distance and alignment; frequency (*f*) and impedance (*Z*) tuning to compensate for changes in system impedances. Impedance tuning has been proposed at the external side (Tx) and/or the implant side (Rx) in several systems.

These adaptive methods are designed with regard to constraints on transcutaneous systems, primarily at the receiver, where tuning must be miniature and low power. Fewer restrictions exist at the transmitter in terms of size, but tuning time and complexity is still a concern. The goal of adaptation also depends on the characteristics of the system, mainly the field region as determined by the operating frequency, antenna sizes, antenna separation and properties of the transmission medium. The recent focus on miniature implants has led to research on focusing fields and midfield operation, achieving efficient power transfer in weakly coupled systems by minimizing tissue absorption, while accounting for implant migration and impedance variations. Challenges addressed in the literature include tradeoffs between tuning time and complexity, coordinating adaptation at the transmitter and receiver and implementing adaptation within power and size constraints at the implant.

Future directions for adaptive transcutaneous device research include addressing the effects of expected variations in the tissue medium, adaptation within power requirements of passive implants and monitoring absorption in tissue to inform adjustments to transmit power, frequency and impedance matching. Variations in the tissue medium are expected among patients, among different areas of the body and over time within a single patient and are therefore expected to contribute to variations in transcutaneous powering of implantable devices. Although progress has already been made on low-power adaptation of implanted devices [90], further efforts to reduce power requirements at the implant will enable passively-powered (battery-less) adaptive implants and, therefore, longer implant lifetime. To continue to address safety issues associated with transcutaneous power, future research will likely continue to address the adaptation of devices operating within SAR limitations. Specifically, methods of detecting SAR or tissue heating would provide another input for evaluating transcutaneous system efficiency in terms of power delivered relative to power dissipated in the tissue.

Implementations of adaptive transcutaneous systems will have substantial effects on implantable medical devices, enabling safer and reliable wireless powering and thereby reducing dependence on implanted batteries, as well as facilitating wireless and remote monitoring via implantable devices. In particular, adaptive devices that are able to compensate for differences in the antenna positioning and system impedances have the potential to provide improved readings from implantable sensors due to more stable powering. Implantable medical devices have already revolutionized treatment and improved quality of life for many patients, and adaptive functionality is positioned to be the next major advance in implantable device technology.

## Figures and Tables

**Table 1:** Summary of literature on adaptive transcutaneous systems.

	Goal	Tuning		Feedback		Variation
		Tx	Rx	Tx	Rx	
[77]	Min reflections	Frequency (VCO)		Reflected voltage		Resonant frequency
[78]	Max efficiency	Frequency (VCO)		Antenna voltage		Resonant frequency
[40]	Max received voltage	Frequency (VCO), Power (supply voltage)			Rectified voltage	Resonant frequency, coupling
	Max driver efficiency	Amplifier $Z_{L}$ (transductor)		Phase between LC and coil driver voltage		Impedance due to frequency change
[82]	Resonance	Impedance (varactor)		Reflected voltage phase change		Distance and impedance
[83]	Resonance	Impedance (duty-cycled C)		Frequency		Impedance
[39]	Stable received power	Power (supply voltage)			Rectified voltage	
[13]	Stable received power	Impedance (duty-cycled C)			Rectified voltage	
[36]	Stable received power	Power (supply voltage)			Storage capacitor voltage	Movement and load impedance
[73]	Max efficiency	Frequency		Resonator voltage		Coupling, impedance
[80]	PA optimal load	Matching impedance (simulated)		Antenna impedance		Impedance
[31,89,93]	Match to PA or LNA	Matching impedance (varactor, switched C bank)		Antenna impedance		Impedance
			Matching impedance (varactor)		Antenna impedance	Impedance
[14,41]	Max power transfer efficiency	Power (supply voltage)			Rectified voltage	Position, fibrous tissue growth
		Impedance			Rectified voltage	
[64,71,81,90]	Max power transfer efficiency		Matching impedance (switched C bank)		Rectified voltage	Position, impedance
		Matching impedance (switched C bank)			Rectified voltage	
[37]	Stable received power	Power (supply voltage)			Rectified voltage	Distance, alignment
[79]	Max received voltage	Impedance (switched C bank)			Rectified voltage	Position
	Stable received power	Power (supply voltage)			Rectified voltage	Position
[72]	Max efficiency (optimum load)		Impedance (switched C bank)		Rectified voltage gradient	Load impedance
[34]	Stable received power	Frequency (ZVS)		Switch transistor drain voltage		Coupling, load impedance
[15]	Max efficiency (relative to absorption)	Field pattern (antenna feeds)			Rectified voltage	Position
[92]	Max power transfer		Impedance (switched CLC pi bank)		Antenna port voltage	Impedance
[43]	Max power transfer	Frequency (ZVS)		Resonant tank voltage		Coupling
[84]	Max efficiency	Impedance (variable C)		S-parameters		Coupling
[23]	Max power transfer	Matching impedance (variable C)		S-parameters		Coupling
	Max efficiency	Power		Reflected voltage		Coupling
[12]	Stable power, max efficiency	Power, resonant frequency			Received power	Coupling
[94]	Resonance	Impedance (switched C)		Output voltage, tuning capacitor voltage		Resonant frequency

## Abbreviations

The following abbreviations are used in this manuscript:

ICD: Implantable cardioverter/defibrillator
VAD: Ventricular assist device
EM: Electromagnetic
FCC: Federal Communications Commission
SAR: Specific absorption rate
MEMS: Microelectromechanical systems
CMOS: Complementary metal-oxide semiconductor
Tx: Transmitter (external)
Rx: Receiver (implanted)
